# Case of Spontaneous Rupture of the Uterus, with Recovery

**Published:** 1880-03

**Authors:** W. D. Hoyt

**Affiliations:** Rome, Ga.


### CASE OF SPONTANEOUS RUPTURE OF THE UTERUS, WITH RECOVERY.

By W. D. HOYT, M.D., Rome, Ga.

On the night of the 3d of January, 1879, I was called to see Maggie Brown, colored, twenty-four years of age, in labor with her fourth child. She was a short, black, stout negress, whom I had known for some years, having been called some years previously to see her, in consultation with another physician, in her first labor. She had then been in labor two days and a night, and it was at a late hour of the second night that I was called to see her. I found her with a very contracted antero-posterior diameter of the superior strait, it being, as nearly as I could ascertain by measuring with my finger, a little less than three inches. The pains were strong but ineffective; the head was partially withdrawn through the strait, and evidently pressing on the sacral nerves. After some time I got the consent of the attending physician to the use of the forceps, and delivered her of a male child of a large size, which was born dead. She suffered greatly from the pressure on the sacral nerves, and was partially lamed in consequence. I attended her alone in her second confinement; and early in the labor, when the head had fairly engaged in the superior strait, I applied the forceps and gradually assisted the labor, making traction efforts only during the occurrence of pains. In this way it took me over an hour to deliver her of another male child of large size. The heart beat for some time after birth, but respiration could not be induced and the child died. The patient recovered satisfactorily from this labor, having only the same amount of lameness as before. Her third child, a female of small size, was delivered spontaneously, under the care of another physician, and lived. I had advised her not to go to full term, but to have labor induced at the seventh or eighth month, which advice was not followed.

It was early in the evening of the 3rd of January when I was called to see her. I found the head presenting in the first position; the pains were strong, and as she had been delivered of one live child, I was disposed to wait and watch the efforts of nature. The waters broke spontaneously, and the contracting process was going on, by which the head was being gradually forced through the superior strait, when suddenly, in the midst of a violent pain, she cried out that she had a severe pain in her stomach. At first, I thought it was only colic, but finding her pains had ceased, I felt her wrist and found there was no perceptible pulse, while her appearance indicated severe shock. Placing my hand over the abdomen, I could feel the buttocks of the child partially protruding, encircled by the tense womb. The friends asked if they should go for another physician. I told them there was no time, and immediately applying my forceps (a pair of John Rohrer's Bettles forceps) I rapidiy delivered the head. There were no pains to assist me, and I had considerable difficulty in delivering the shoulders; but with my fingers in the axilla I delivered first the right and then the left shoulder. As the last shoulder escaped from the vulva there came a gush and the rest of the chi.d's body, with the after birth, and clotted and liquid blood, all came away together, sprinkling blood in every direction. It was evident that the placenta was completely separated, and was lying loose in the womb. The womb now contracted well and the pulse returned. Giving my patient full doses of opium and making her as comfortable as circumstances admitted, I turned my attention to the child. I found a female child, weighing, I suppose, eight or nine pounds, which I failed to resuscitate. As I desired to remain near my patient, I laid down, but about 3 A. M. was called to see her. I found her in great pain, and as there was very little discharge, the supposition was that clots had formed in the womb; I introduced my hand for the purpose of ascertaining. I found there were only a few small clots, but felt the rent, through which I could pass my hand. It is my impression that the peritoneal investment of the womb was not torn. The womb seemed thinned at the point of laceration, and had a harsh, degenerated feel. The pains proceeded from a small hour-glass contraction to the left of the rent, in which a small clot was included. Dilating the contractions and turning out the clot stopped the pains. After this she rested comfortably until morning.

Thə following are the notes of the case :—

4th. 5.30 P. M. Pulse 126; temperature 99.2°.

5th. Passed a good night until 3 A. M. Passed water twice, early in the night and at 4 A. M. Taking light nourishment; abdomen tympanitic. 2 P. M. Pulse 124; temperature 101°

6th. Had pains through the night; slept toward morning; meteorism moderate; lochia pale; urine dribbling away; took some nourishment. 4 P. M. Pulse 122; temperature 103.2°.

7th. Slept well; considerable perspiration; has a fair appetite; soreness diminished; urine dribbling away; no collections in the bladder. 1 P. M., pulse 102, temperature 98.6.

8th. Pains through the night; bowels acted spontaneously; passed urine; tympanitis dimished; womb less engorged; appetite good. 5 P. M., pulse 104, temperature normal.

10th. Slept well; bowels moved several times; the actions at first dark and now yellow; milk came last night; soreness of abdomen dimished. 1 P. M., pulse 116, feeble, temperature 100.8.

12th. Sciatic pains night before last; the bowels are regular; the appetite good; the milk is dried up; she feels well except for the sciatic pains. 2 P. M., pulse 100, soft, temperature 100.2.

15th. Doing well, but complains of sciatic pains. Pulse 100, soft, temperature normal.

16th. Had chills and fever last night, which I judge to be malarial. Pulse 110, temperature normal.

*Treatment.*—For days the patient was kept under the inflnence of opium, administered every two to four hours when awake ; quinine was given from the start, ten grains being given between 12 and 6 A. M., and the same quantity between 12 and 6 P. M. When her fever rose to 103 the quantity was increased to I5 grains ; on the occurrence of the chill some calomel was given and the quinine continued. Iodide of potassium and Fowler's solution were used for the sciatic pains. With the view of filtering zymotic germs from the air penetrating the womb, a pledget of cotton was kept constantly applied to the vulva.

After the last record made she progressed favorably, and made a good recovery. I now frequently see her on the street, in good health.—*Philadelphia Reporter.*

